# Diminished Expression of Fat and Dachsous PCP Proteins Impaired Centriole Planar Polarization in Drosophila

**DOI:** 10.3389/fgene.2019.00328

**Published:** 2019-04-12

**Authors:** Sergio Garrido-Jimenez, Angel-Carlos Roman, Jose Maria Carvajal-Gonzalez

**Affiliations:** ^1^Departamento de Bioquímica, Biología Molecular y Genética, Facultad de Ciencias, Universidad de Extremadura, Badajoz, Spain; ^2^Champalimaud Neuroscience Programme, Lisbon, Portugal

**Keywords:** centrioles, planar cell polarity, fat and dachsous, actin polymerization, planar cell polarity effectors

## Abstract

Proper ciliary basal body positioning within a cell is key for cilia functioning. Centriole and basal body positioning depends on signaling pathways such as the planar cell polarity pathway (PCP) governed by Frizzled (Fz-PCP). There have been described two PCP pathways controlled by different protein complexes, the Frizzled-PCP and the Fat-PCP pathway. Centriole planar polarization in non-dividing cells is a dynamic process that depends on the Fz-PCP pathway to properly occur during development from flies to humans. However, the function of the Ft-PCP pathway in centrioles polarization is elusive. Here, we present a descriptive initial analysis of centrioles polarization in Fat-PCP loss of function (LOF) conditions. We found that Fat (Ft) and Dachsous (Ds) LOF showed a marked centrioles polarization defect similar to what we have previously reported in Fz-PCP alterations. Altogether, our data suggest that centriole planar polarization in Drosophila wings depends on both Ft-PCP and Fz-PCP pathways. Further analyses in single and double mutant conditions will be required to address the functional connection between PCP and centriole polarization in flies.

## Introduction

Cilium is an organelle projected at the cell surface with several crucial functions during development and in adult organs. Cells use their cilia to communicate with the environment or to physically interact with the surrounding media (Satir and Christensen, 2007; Goetz and Anderson, 2010; Ishikawa and Marshall, 2011; Malicki and Johnson, 2017). An example of signaling associated with cilia is its vastly known function on Sonic hedgehog signaling (Briscoe and Therond, 2013). On the other hand, a mechanical function for cilia could be found in the choroid plexus lining the brain ventricles, where the beating of the cilium at their apical membranes generates the proper directional fluid flow of the cerebrospinal fluid (CSF) (Brooks and Wallingford, 2014). Based on these two types of cilia functions (signaling and/or mechanical), many genetic disorders or diseases underlying cilia miss-functioning have been described. These diseases are included and named as ciliopathies, and most of them are initiated during early development (Hildebrandt et al., 2011; Novarino et al., 2011; Braun and Hildebrandt, 2017; Reiter and Leroux, 2017).

Structurally, cilia consist of membrane surrounding a cytoskeletal structure known as the axoneme, which is anchored to the basal bodies. In ciliopathies, cilium miss-functioning is usually associated to defective assembly or placement in a group of cells (Hildebrandt et al., 2011; Braun and Hildebrandt, 2017). Cilia assembly is controlled by structural proteins linked to the cytoskeleton like tubulin but also to trafficking related proteins and organelles like endosomes (Bernabe-Rubio and Alonso, 2017; Mirvis et al., 2018). Cilia positioning is also associated to the cytoskeleton, but in this case, it depends on the cell polarity pathways controlling the three-dimensional distribution of organelles and plasma membrane composition at the single cell level. In polarized epithelial cells, cilia are projected from the apical membrane and in some specialized tissues are restricted to sub-areas at that apical membrane, named translational polarity, and/or their basal bodies orient their basal foot to the same direction, named rotational polarity (Carvajal-Gonzalez et al., 2016a; Adler and Wallingford, 2017).

This positioning of cilia needs also to be coordinated within the tissue to produce a global response like coordinated ciliary beating. A key pathway involved in coordinating cells during tissue development is the Planar cell polarity (PCP) pathway, a conserved pathway from Drosophila to humans. PCP is established and maintained by two signaling cascades, the Frizzled PCP (Fz-PCP) and the Fat PCP (Ft-PCP) pathways. Fz-PCP pathway is governed by two protein complexes, the Frizzled/Disheveled/Flamingo/Diego (Fz/Dsh/Fmi/Dgo) complex and Vang/Prickle/Flamingo (Vang/Pk/Fmi) complex (Goodrich and Strutt, 2011; Adler, 2012; Peng and Axelrod, 2012; Singh and Mlodzik, 2012; Carvajal-Gonzalez and Mlodzik, 2014; Devenport, 2014). The Ft-PCP pathway is based on the interaction between Fat (Ft) and Dachsous (Ds). The interaction across adjacent membranes of these proto-cadherins is coordinated by the Golgi resident kinase Four-jointed (Fj) (Zeidler et al., 1999, 2000; Matakatsu and Blair, 2004, 2006; Simon, 2004; Brittle et al., 2010, 2012; Simon et al., 2010; Mao et al., 2011). Fz-PCP has been shown to control the orientation of the cilia by controlling the docking of the basal body including the mother and daughter centrioles. This connection between Fz-PCP and basal bodies/centriole positioning is a well-conserved function in different organs across species. On the contrary, the function of Ft-PCP in ciliary basal bodies/centriole positioning is not clarified yet. It has been shown that in *Fat4* knock-out mice, hair cells are mis-orientated throughout the cochlea when looking at stereocilia (Saburi et al., 2008), however, the kinocilium basal body positioning was not assessed.

We have recently found that centriole positioning was also controlled by the Fz-PCP pathway in Drosophila epithelial cells where cilia are absent (Carvajal-Gonzalez et al., 2016a,b). In Drosophila pupal wings, we were able to show that centrioles polarization (off-centered movement of centrioles at the apical planes) was a dynamic process that occurs during trichomes formation, and that this polarization was abnormal when Fz-PCP signaling was defective using loss or gain of function experimental conditions (Carvajal-Gonzalez et al., 2016a,b). Since Ft-PCP is also important during morphogenesis in Drosophila pupal wings, here we decided to test the connection between Ft-PCP and centriole positioning in this model system.

## Materials and Methods

### Fly Strains

**FIGURE 1:**
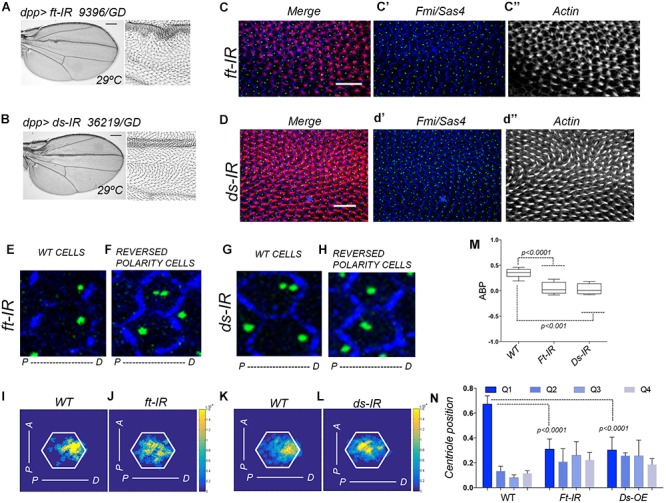
Ft-PCP pathway LOF conditions affect centriole polarity. **(A,B)** In adult wings, single knock-down for Ft or Ds using *dpp>ft-IR or dpp>ds-IR* generates hair mis-orientation phenotypes and rounded wing shape. Both phenotypes have been previously observed in Ft and Ds mutants. **(C,D)** In *dpp>ft-IR or dpp>ds-IR* pupal wing hair mis-orientation phenotype are also presented in the dpp domain (actin-based hair labeled with phalloidin in red, *Sas4* coupled to GFP in green to mark centrioles, *Fmi* in blue for cell junctions). **(E–H)** Cells with WT orientation of hairs showed proper centriole localization at the distal side of the cell **(E,G)**. On the other hand, neighbor cells knocked-down for Ft or Ds with reversed polarity (reversed hair orientation) did not localize centrioles to the distal side of the cell **(F,H)**. **(I–L)** Centriole distribution visualization in density maps showed that both, *dpp>ds-IR* and *dpp>ft-IR*, centriole positioning is affected when compare to WT areas of the same wings. **(M,N)** Quantification of centriole polarization using the ABP method **(M)** or the Q method **(N)** showed that cells knocked-down for Ft or Ds failed to polarized centriole to the level of WT cells. Statistical analyses among experimental groups: *t*-test. Scale bars in **(A,B)** represent 25 mm Scale bars in **(C,D)** represent 10 μm.

To analyze the role of Ft-PCP in centriole polarization in Drosophila pupal wings we first tested all the available RNAi lines targeting the known components of the Fat-PCP pathway including, Fat (Ft), Dachsous (Ds), Dachs and Four jointed (Fj). All culture and cross of fly lines were performed on standard medium and maintained at indicated temperatures (25 or 29°C). Following fly lines were used in this study: Ft-IR (9396/GD and 108863/KK VDRC stocks), Ds-IR (36219/DG and 4316/GD VDRC stocks), Dachs-IR (12555/GD; 32142/GD and 102504/GD VDRC stocks), Fj-IR (6774/GD VDRC stock). GAL4/UAS system was used to direct UAS-RNAi constructs to *decapentaplegic* (*dpp*) wing compartment, a stripe between L3 and L4. In our experimental conditions, we found that only two lines (Ft and Ds) produced robust PCP phenotypes (Figure 1A,B and Supplementary Figure S1F). These phenotypes in adult wings corresponded to hair mis-orientation in the proximal part of the wing near the L3 vein (Figure 1A,B). None of the tested lines for Fj generates PCP phenotypes under our experimental conditions (Supplementary Figures S1D,E) and out of the three lines tested for Dachs (Supplementary Figures S1A–C), only 1 line showed hair mis-orientation phenotypes but not in all the wings analyzed (Supplementary Figure S1C).

### Adult Wing Analyses

For adult trichomes analyses, wings were removed, washed in PBS 0.1% Triton X-100 (PBS-t) and mounted on a slide in 80% glycerol in PBS. Adult wings were imaged on a BX51 direct microscope (Olympus). Images acquisition was performed with a camera (DP72, Olympus) and CellD software (Olympus).

### Immunohistochemistry

Fly pupae at white stage were collected and cultured at 29°C for 25 or 28.5 h. Wings were dissected in PBS-t and fixed for 1 h with 4% paraformaldehyde (PFA). Pupae were washed in PBS-t three times for 5 min and blocked in PBS-t with 2% bovine serum albumin (BSA) for 45 min. Dissected pupae were incubated overnight with primary antibody at room temperature in PBS-t-BSA. After incubation, samples were washed five times in PBS-t and incubated in fluorescent phalloidin and fluorescent secondary antibodies for 90 min, both diluted in PBS-t-BSA. Five additional washes were performed in PBS-t, and pupal wings were detached from the pupal cage and mounted on slides with medium for fluorescence (Vectashield, Vector Laboratories). To stain the cellular membrane was used anti-Fmi (from DSHB). Secondary antibody conjugated with the fluorophore Alexa-405 (Invitrogen) and Alexa 594-phalloidin (Invitrogen) were used at 1:200.

### Image Acquisition and Processing

To orientate pupal wings, the distal part was always pointed to the right side. Images were acquired using FV 1000 confocal microscope (Olympus). After acquisition, images were processed using Fiji, to generate the cell borders mask (Tissue Analyzer plugin), and Adobe Photoshop CC 2015 to create the color-coded mask based on the phenotype.

### Heat (Density) Maps

We used density maps in order to visualize relative centriole positioning from a set of cells. Briefly, we modeled each cell to a regular hexagon with its area being the pixel numbers of the cell, and then we normalized all the modeled hexagon cells to the same size. Afterward, we normalized the position of the centriole relative to the centroid of its cell to the model cell. We have found that every cell in the Drosophila pupal wing has two centrioles but in many cases these two centrioles are too close to be separated, many times they are sitting one on top of the other. Our method compute each centriole individually. Finally, we represented the centriole density in the set of cells, so each pixel in the density map represents the probability of finding a centriole in that set of cells.

### ABP Method

Average Basal Body Position (ABP) for each sample was calculated following Hashimoto et al. (2010) protocol. Briefly, the score of each cell represented the normalized position of the centriole along the anterior-posterior axis (being - 1.0 the minimal anterior coordinate of the cell, 0 the cell centroid and 1.0 the maximal posterior coordinate of the cell). Then, the ABP score was calculated as the average value of the cells in an image.

### Q Method

Each centriole was assigned to a specific Quartile (Q) depending on their relative position to the anterior-posterior axis of the cell that crosses the cell centroid (Taniguchi et al., 2011). If the centriole was located between -π/4 and π/4, then it was assigned to Q1. If the centriole was located between π/4 and 3π/4, it was assigned to Q2. In the case of being located between -π/4 and -3π/4, then it was assigned to Q3, and finally the rest of centrioles were assigned to Q4.

### Statistical Analyses

Data were analyzed using *t*-test (GraphPad Prism) for Q and ABP methods.

## Results

We have previously described that centrioles polarization is a dynamic process that occurred during hair formation in Drosophila pupal wings. In wild type (WT) conditions, centrioles move from centered positions at the most apical part of pupal wing epithelial cells and as the actin-based hair is forming centrioles move off center toward the base of the hairs (Carvajal-Gonzalez et al., 2016b; Garrido-Jimenez et al., 2018). To analyze the polarization of centrioles in Ft-PCP deficient conditions, we combined hair staining with phalloidin to assess those wings with well-formed hairs, while using GFP-Sas4 fluorescent expression to visualize centrioles and immunolabeled Fmi to delimit each epithelial cell (Figure 1C–H for higher magnifications). To visualize the centriole population distribution, we employed a heat (density) map for a WT or its RNAi neighbor field (Figure 1I–L). To properly quantify centriole polarization, we used two different standard methods, the ABP method and the Q method. Briefly, the ABP method measures the anterior or posterior positions of centrioles relative to the center of the cell (visualized from the top). On the other hand, the Q method quantify the distribution of centrioles in 45 degrees’ quartiles (Q1, Q2, Q3, and Q4). In regions with hair mis-orientation phenotypes generated by knock-down of Ft or Ds we found that centrioles fail to properly polarize toward the distal side at the apical planes, as shown in density maps for Ds or Ft knocked-down cells compared to near WT cells (Figure 1I–L). Looking at the Q1-4 values, similar percentages of centrioles are found in each of them in Ft-IR and Ds-IR conditions. In contrast above 60% of centrioles are found in Q1 for WT cells (Figure 1N). In addition, the ABP quantification in Ft-IR and Ds-IR conditions showed that centriole remained at more centered position in the cell when compared to centriole polarization in WT areas of the same wings (Figure 1M). Altogether, these results support the hypothesis that centriole polarization is also controlled by the Fat and Dachsous PCP pathway.

## Discussion

This article together with the previously published data on cilia and PCP in vertebrates pushes forward the idea that PCP is a well-conserved regulator of centriole positioning. It also highlighted for the first time the importance of Ft-PCP in centriole positioning independently of cell division. In vertebrates, there are four Fat homologs (Fat1–4), two Ds homologs (Ds1 and Ds2), and one Fj ortholog (Fjx1) (Rock et al., 2005). *Fat4* knock-out mice have been described to have dilated tubules and cysts formation in their kidneys. This phenotype is connected to randomization of spindle orientation affecting oriented cell division, which is important for tubule elongation and single lumen formation (Saburi et al., 2008). In addition, it has been shown that PCP proteins such as Fat4 and Vangl2 localize to the base of cilia in cultured cells. Interestingly, Fat4 is localized to the primary cilium. Furthermore, Dchs1 knock-out mice showed mild defects in early uroteric bud branching morphogenesis, resulting in kidneys that are reduced in size (Mao et al., 2011).

**FIGURE 2:**
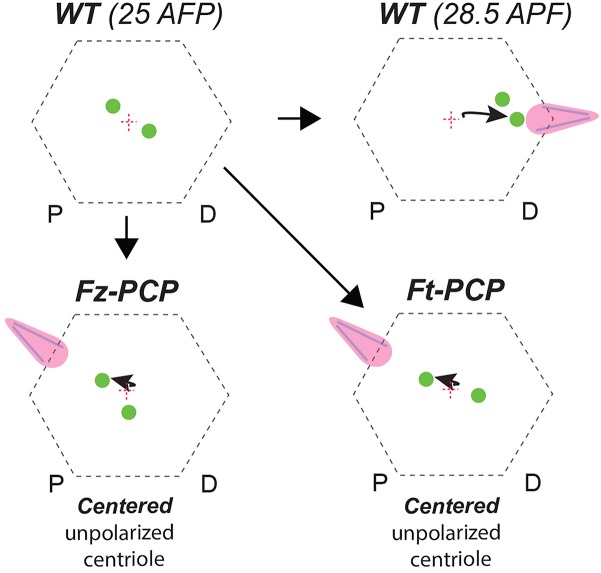
Centriole positioning illustration in WT and Fz-PCP or Ft-PCP deficient conditions. Schematic representation of the phenotypes described. In WT Drosophila wing epithelial cells aged to 28.5 APF (after puparium formation) centrioles migrate to base of the actin-hairs. Under the same experimental conditions, in Ft-PCP LOF conditions centrioles failed in this polarized movement. This phenotype resembled the one found in Fz-PCP LOF conditions already published (Carvajal-Gonzalez et al., 2016b).

Moreover, over-expression of the Sple isoform of *pk* in developing wings has recently been reported to reverse PCP orientation, resulting in actin hair formation being moved to the proximal cellular vertex without affecting Vang or Dsh localization (Ayukawa et al., 2014; Olofsson et al., 2014). We have previously published that Pk over-expression in Drosophila pupal wings cause centriole positioning defects concomitant with reversed actin-based hair formation (Carvajal-Gonzalez et al., 2016a,b). In addition, Sple-OE was shown to modulate coupling between the Fz-PCP and Ft-PCP pathway (Merkel et al., 2014). As a whole, we can envision a system where centriole positioning is a global output controlled by both PCP pathways (Figure 2). Hence, this new role for Ft-PCP could be linked to Fz-PCP rather than independent of it. Future experiments are required to confirm or rule out this long-standing conflict about the interdependency of these two PCP pathways.

## Author Contributions

SG-J performed all of the experiments. A-CR designed and performed the data analysis. JC-G designed the experiments, analyzed data, and wrote the manuscript.

## Conflict of Interest Statement

The authors declare that the research was conducted in the absence of any commercial or financial relationships that could be construed as a potential conflict of interest.

## Supplementary Material

FIGURE S1 Fat-PCP pathway related RNAi lines used in adult screening that do not cause strong hair mis-orientation phenotypes. **(A–F)** Images from adult wing aged at 29°C and knock-down for single Ft-PCP components using *dpp>gal4 as a driver* that do not generate hair mis-orientation phenotypes. Scale bars represent 250 mm.
