# Supplementary figures and images for: Diminished Expression of Fat and Dachsous PCP Proteins Impaired Centriole Planar Polarization in Drosophila
Source: Front Genet. 2019 Apr 12;10:328. doi: 10.3389/fgene.2019.00328 (PMC6473044; doi:10.3389/fgene.2019.00328)

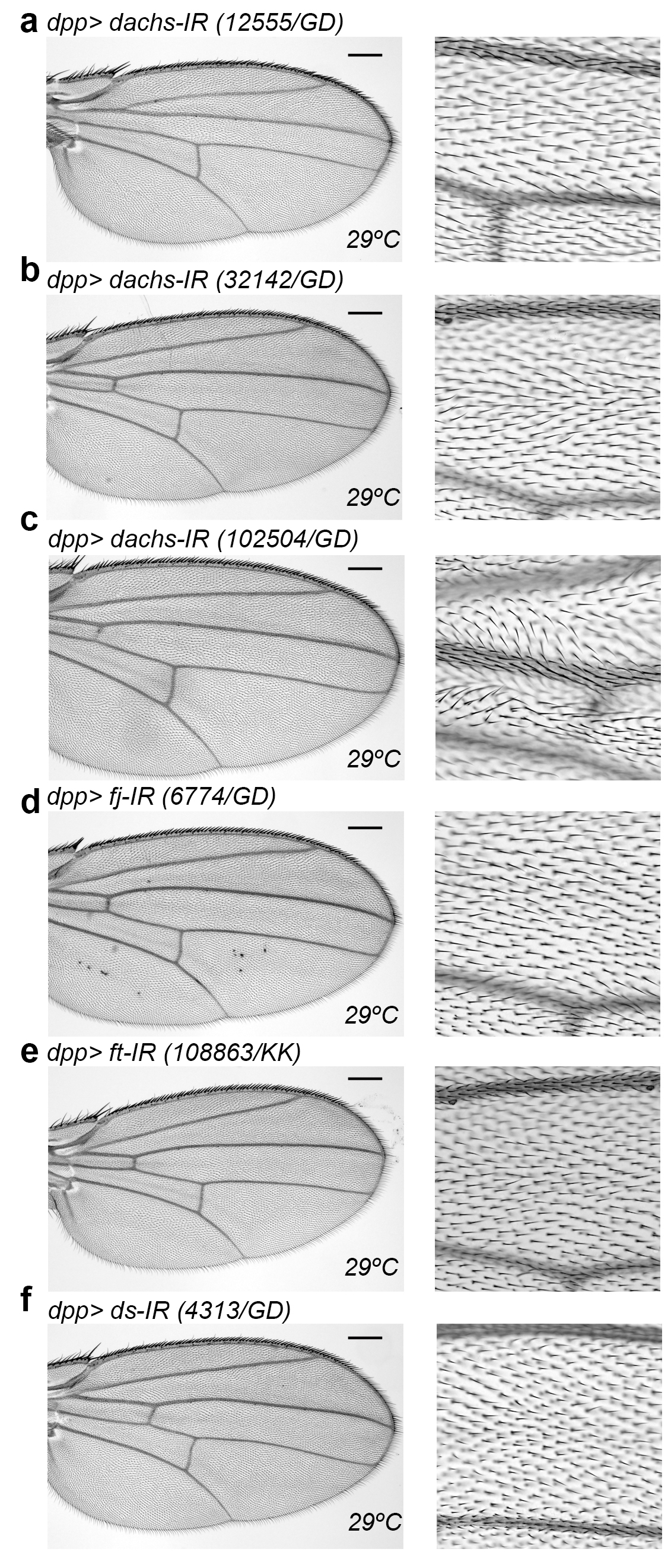

Supplement: FIGURE S1 — Fat-PCP pathway related RNAi lines used in adult screening that do not cause strong hair mis-orientation phenotypes. (A–F) Images from adult wing aged at 29°C and knock-down for single Ft-PCP components using dpp>gal4 as a driver that do not generate hair mis-orientation phenotypes. Scale bars represent 250 mm. [file Image_1.JPEG]
